# Quantitative analysis of new drug registration policies for traditional Chinese medicine using the PMC index model

**DOI:** 10.3389/fpubh.2025.1585350

**Published:** 2025-05-27

**Authors:** Liu Yiming, Xie Ming

**Affiliations:** Liaoning University of Traditional Chinese Medicine, Dalian, China

**Keywords:** PMC index model, Chinese medicine registration policy, quantitative evaluation study, policy optimization, China, traditional Chinese medicine

## Abstract

**Introduction:**

Traditional Chinese medicine (TCM) culture represents the essence of traditional Chinese culture. The registration of TCM is crucial for promoting its sustainable development and can significantly contribute to the economic growth of the TCM industry. Consequently, the Chinese government has introduced a series of policies regarding TCM registration, aiming to provide scientific and effective guidance for the innovation, inheritance, and development of TCM. At present, China’s TCM registration policies are still inadequate, and it is necessary to evaluate and compare them quantitatively.

**Methods:**

The present study utilizes text mining methodology and the Policy Modeling Consistency Index (PMC index) model to establish an evaluation system for quantitative assessment and comparison of the Drug Administration Law, Drug Registration Administration Method, Chinese Medicine Registration Administration Method, Chinese Medicine Law, and other relevant legislations spanning from 1985 to 2023.

**Results:**

This study revealed that (1) through a comprehensive analysis of high-frequency words, different Traditional Chinese Medicine Regulatory Policies (TCMRPs) shared similar content and objectives, but placed varying degrees of emphasis on specific aspects. (2) The average PMC index of 165 TCMRPs was 5.858, which generally fell within the acceptable range. Among these policies, none achieved perfection, while 39 policies were deemed excellent, constituting 23.6% of the total policies. There were a total of 119 policies falling within the acceptable range, accounting for 72.1% of the total. Additionally, there were 7 policies with below-standard performance, making up for only 4.2% of the total. (3) The PMC index values differed significantly across issuing institutions and incentive methods for various TCMRPs, with generally low scores observed in this regard. However, there were similarities in terms of policy nature, timelines, function, content, mode and scientism rating among these policies.

**Conclusion:**

(1) The TCM registration system requires further enhancement and refinement to ensure greater efficiency and compliance with current standards. (2) Technical problems have hindered the research and development of TCM. (3) Talent preservation should be incorporated as a key consideration in the formulation of TCM registration policies. (4) The formulation of TCM registration policies should incorporate economic incentive considerations. (5) China ought to intensify the development of classic and renowned formulas to expedite the registration of novel TCM.

## 1 Introduction

The development of traditional Chinese medicine (TCM) is highly valued by the CPC Central Committee and the State Council, as evidenced by the issuance of the Opinions on Promoting the Inheritance, Innovation, and Development of TCM (hereinafter referred to as the “Opinions”). These Opinions clearly articulate a strategic approach toward promoting medical inheritance and innovative development, while also employing modern scientific interpretations of TCM principles (1). The State Drug Administration has fully implemented the directives outlined in the “Opinions” by integrating scientific and technological advancements, actively fostering supervision science within TCM, and reforming and enhancing review processes for batches of Chinese medicines. Furthermore, the General Office of the State Council has issued policy measures aimed at accelerating TCM’s characteristic development. These measures optimize various policies related to reviewing and approving Chinese medicines, ensuring that qualified new products can enter an expedited review and approval pathway (2). TCM plays a crucial role in supporting the clinical treatment of diseases and ensuring human health. Additionally, TCM continues to innovate in its inheritance and development, promoting mutual progress. (1) Innovative TCM is an integral part of the pharmaceutical industry with immense potential for future advancements that will bring significant economic and social benefits to the country (3). (2) Artemisinin, a compound extracted from TCM, has played a crucial role in addressing the global challenge of malaria. Moreover, TCM has played a vital role during the COVID-19 pandemic. Cancer and autoimmune diseases remain unsolved health challenges worldwide. However, research indicates that innovative drugs have shown promising results in improving cure rates for these conditions (4). (3) By 2023, the global innovative drug market has been expected to reach $1,034.5 billion, accounting for about 70% of the total global pharmaceutical market. China has a smaller share of the innovative drug market, accounting for only about 12.5%, of which TCM innovative drugs account for a smaller proportion. After nearly two decades of development, since 2014, China has become the world’s second-largest pharmaceutical market after the United States. According to an IQVIA report, China’s pharmaceutical spending growth over the past 5 years (2018–2022) was primarily driven by innovative drugs, averaging 8.5% annual growth (5, 6). The market share of original drugs increased from 20% in 2014 to 29% in 2023. Only about 7.9 percent of innovative drugs in China are approved by the FDA after Phase I clinical trials. The average development cost of innovative drugs in 2021 will reach $2.006 billion, with an average development time of approximately 6.9 years (7, 8). The research and development (R&D) trajectory of innovative TCM presents significantly greater methodological and regulatory challenges compared to conventional herbal medicinal products. The characteristics of high investment and low success rate lead to the risk aversion of new drug research and development. Consequently, the predominant focus of pharmaceutical enterprises on generic drug research has inadvertently constrained their innovative development. Under the favorable national policy, Chinese medicine enterprises are at a turning point. However, research and development in the industry still seems to be stagnating (9–11).

The TCM registration policy encompasses the Drug Administration Law, guidelines, implementation opinions, supplementary regulations, and the TCM Law. The government has placed significant emphasis on the innovation and development of TCM culture and actively promoted the advancement of the TCM industry (12, 13). However, there are variations among different TCM registration policies which make it challenging to determine their overall quality and characteristics. Consequently, it becomes difficult to clearly define the strengths and weaknesses of these policies’ design. As a result, experts and scholars struggle to provide effective targeted improvement measures. Therefore, this paper aims to conduct a quantitative study on Chinese TCM registration policies in order to identify their advantages and disadvantages accurately (14). This will assist decision-makers in establishing better TCM registration policies that promote both the registration process itself as well as the inheritance and innovation of TCM. To achieve this objective, we adopt the PMC index model which is a quantitative policy evaluation method proposed by Estrada in 2011 (15). This model evaluates policy internal consistency from various dimensions enabling us to precisely determine each policy’s strengths and weaknesses while remaining at the forefront of global policy evaluation practices.

“The literature examined in this study commences with the enactment of China’s first Drug Administration Law in 1985, which marked the establishment of legal regulations governing drug supervision and administration (16). Notably, the issuance of specialized regulations on the registration and administration of TCM by 2023 signifies an increasing recognition of TCM’s status and characteristics, as well as a commitment to its development. The key contributions of this study are twofold: (1) Collecting and organizing pertinent legal documents on drug registration issued in China from 1985 to 2023, excluding chemical drug registration and biological drug registration that lie beyond the scope of this investigation. Ultimately, we selected 165 policies specifically related to TCM registration for analysis. (2) While existing literature has conducted quantitative analyses on general drug registration policies, there has been a lack of systematic analysis solely focused on TCM registration. Moreover, most current studies evaluate policy through macro analysis without delving into its content. Therefore, our study employs text mining methods to delve into the textual aspects of TCM registration policy and uncover its underlying fundamental elements and implementation logic. Additionally, we establish the PMC index model for quantitative evaluation purposes regarding TCMRPs while providing theoretical support for future formulation and modification efforts.”

**Figure 1:**
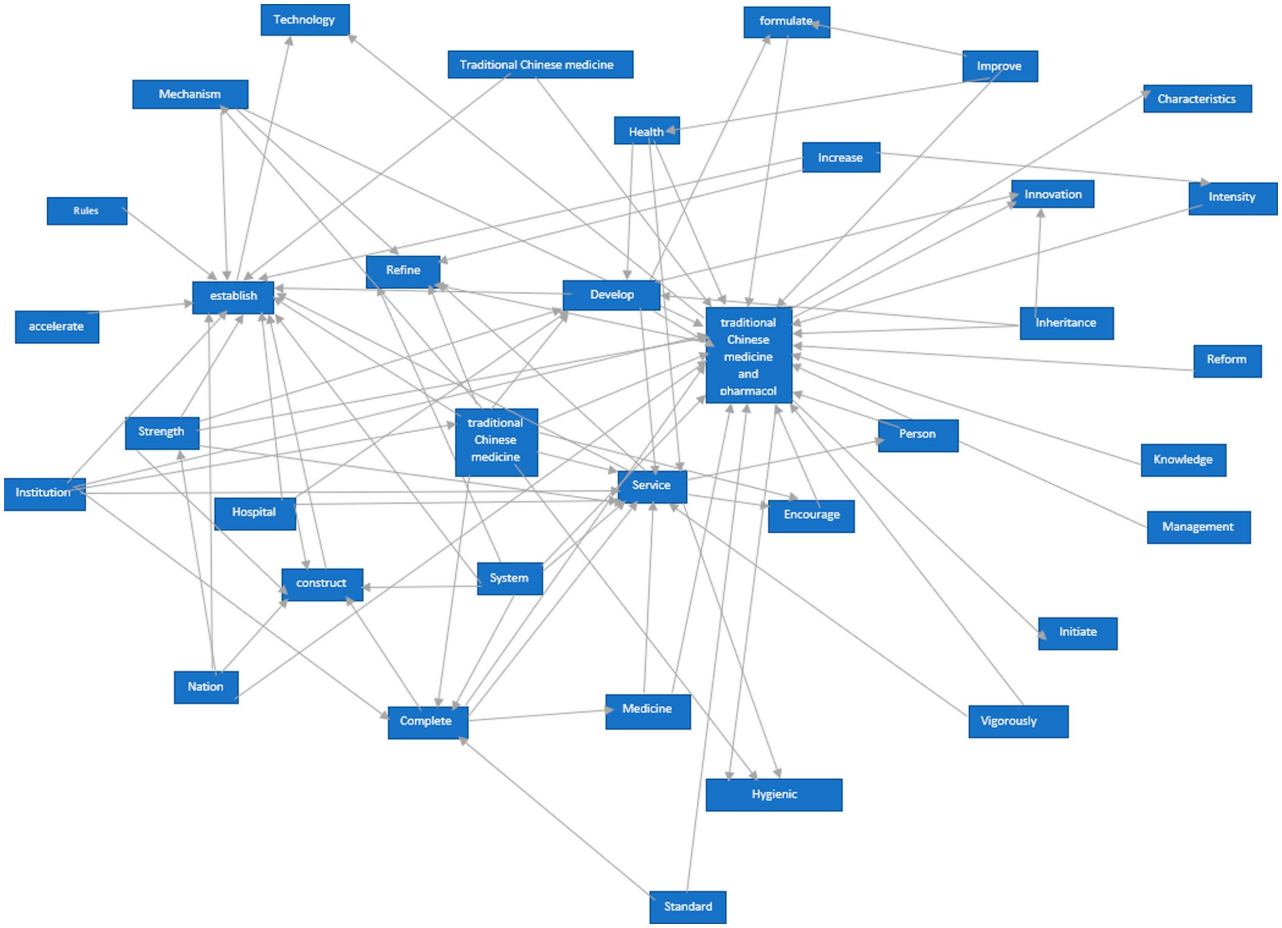
TCMRP network semantic analysis graph.

In conclusion, the present study is divided into several sections. Section 2 provides a comprehensive review of the existing literature on drug registration and policy within the context of TCM, highlighting research gaps that currently exist. In Section 3, we outline the research objectives, methodology, and criteria for sample selection. Section 4 presents the findings from our quantitative analysis of TCMRPs, discussing both their strengths and weaknesses while offering suggestions for improvement (Figure 1). Finally, in Section 5, we summarize our key findings and acknowledge any limitations inherent in this study.

## 2 Literature review

The drug registration process in the United States has been established for 50 years, which is significantly longer compared to most other countries where it typically takes around 25–30 years (17, 18). The success of a national new drug application heavily relies on the pharmaceutical industry representatives’ familiarity and attentiveness toward the specific requirements set by regulatory bodies. International registration documents are formulated based on increasingly standardized requirements and guidelines from regulatory authorities, as well as through closely coordinated efforts within the industry. These documents aim to facilitate enhanced communication and cooperation among regulatory authorities (19). In his research, Florian Naudet discovered that the centralization of multiple administrative registered drugs represents an innovative approach to drug registration, facilitating recognition and coordination among various health authorities. As a result, the EMA and FDA have initiated a collaborative agreement in drug development (20–24). Through literature search, it has been identified that the quantitative research on drug registration policy primarily focuses on evaluating the policy’s implementation effectiveness, while qualitative research predominantly centers around assessing the policy’s content. Among these studies, there is a greater abundance of quantitative research examining the implementation effects of policies, with fewer investigations into the policy content itself. In summary, scholars exhibit significant interest in understanding the current status of drug registration policy implementation (25, 26). “The evaluation of applications by national Competent Authorities (NCAs) at the national level and the evaluation of EU herbal medicinal products (HMPs) by the Herbal Products Committee (HMPC) at the European level constitute the regulatory framework for HMPs in Europe, known as the European HMP regulatory framework. The EU Herbal Medicine monograph, representing HMPC’s scientific view on safety and efficacy, plays a constitutional role in Traditional Use Registrations (TURs), Well-Established Use Marketing Authorizations (WEU-MAs), and marketing procedures within Member States. In conclusion, this robust European framework facilitates harmonization of scientific assessment and promotion of product marketing. For pharmaceutical companies operating outside the EU, leveraging the EU herbal monograph in their European marketing procedures can yield significant benefits. Moreover, this model serves as an exemplary reference for countries and regions outside the EU seeking to establish legislation promoting safe use of traditional Chinese medicine (1). For instance, Daoran Lu et al. examined the impact of the drug registration classification system and the review and approval process on drug innovation and development. They found that these factors have significant implications in this regard. In response to this, China has implemented a series of policies and regulations aimed at reforming the major registration classification system, prioritizing clinical value orientation, and establishing an evaluation system tailored to TCM characteristics. These initiatives are expected to actively foster the advancement of new TCM.” The Food and Drug Administration (FDA) has granted accelerated approval for the utilization of real-world data to simulate post-approval confirmatory clinical trials of therapeutic drugs. This cross-sectional study aims to investigate the feasibility of using real-world data, including billing, claims, and electronic health records, in modeling the confirmatory clinical trials required by the FDA for 50 newly approved therapeutic drugs between 2009 and 2018 (27). According to the research conducted by Shilin Chen, China has implemented simplified registration and approval management regulations for compound TCM prescriptions. Streamlining the registration and approval process for ancient classic prescriptions has expedited the inclusion of new Chinese herbal medicines in the market, reduced research and development costs, and alleviated medical burdens (28). By employing descriptive statistical methods, Xian Su conducted an analysis and processing of the data from clinical drug trials across all exhibition halls, encompassing both suspended and non-suspended trials. The findings revealed a consistent increase in the number and proportion of clinical trials being suspended due to safety concerns over time, with a significant portion still experiencing suspension. Factors such as protocols, researchers/research institutions, and drug availability were identified as the primary reasons for these suspensions. This study holds immense significance in enhancing the dynamic management capabilities of drug registration clinical trials while also providing valuable insights for designing an evaluation system for trial suspensions. Revised sentence: In summary, the aforementioned literature facilitates researchers in exploring drug registration policies from diverse perspectives and offers insights into policy optimization (29). However, these studies primarily examine the macro-level drug registration policy, with a lack of systematic evaluation on TCM registration policy in China. Therefore, further research is needed to investigate the TCM registration policy in China.

Policy evaluation plays a pivotal role in policy formulation and optimization, necessitating a comprehensive and rational procedure. To conduct effective policy evaluation, it is imperative to select an appropriate and scientific assessment method. The prevailing studies commonly employ the following assessment methods: five assessment tools (30), the “3e” assessment framework (31), the Law Change index (32), the analytic hierarchy process (33), the Delphi method (34), content analysis (35), differences in difference analysis (36–38), etc. As previously mentioned, the five assessment tools, the “3e” assessment framework, and the legal change indicator are relatively outdated and one-sided when evaluating policies. Furthermore, AHP, Delphi method, and content analysis entail more subjective evaluation processes (39). Additionally, differences in difference analysis primarily focus on assessing the implementation effect of specific policies (40–42) while lacking systematic reviews of a range of policies (43). Although these aforementioned policy evaluation methods are widely utilized, they possess shortcomings regarding objectivity and accuracy. Moreover, these methods pay insufficient attention to individual variances and policy texts. In contrast, the PMC index model integrates qualitative and quantitative approaches that offer greater comprehensiveness and objectivity since it not only evaluates policy consistency but also systematically analyzes variations among individual policies across multiple dimensions, hence its widespread adoption in policy evaluation (44). The PMC index model was employed by Chengning Yang to analyze China’s adolescent mental health policy, revealing significant deficiencies in the policy-making process. These include a lack of advocacy and supervision policies, an emphasis on short- to medium-term effects, and inadequate comprehensive planning, all of which impede its implementation speed (45). The study conducted by Dai S on China’s ecological protection compensation policy, based on the PMC index model (55), revealed certain limitations in the policy. These include evident internal differentiation, limited effectiveness, and inadequate incentives and guarantees (56). In addition, numerous studies have employed the PMC index model for conducting research on policy evaluation, encompassing health promotion policies (15), TCM development policies (44), green economy development policies (46), public health emergency policies (47), and more. These studies demonstrate that the PMC index model yields favorable outcomes in policy formulation and optimization while possessing robust scientific capabilities for policy evaluation.

In conclusion, the literature on drug registration and policy evaluation is extensive, yet the existing research remains insufficient. Firstly, there is a plethora of literature available on the implementation effects of drug registration policies. However, there are few studies that evaluate these policies from a policy-making perspective. Secondly, there is a dearth of literature regarding TCM registration policies in terms of both policy outcomes and content evaluations. Furthermore, scientific policy evaluation in TCM registration research currently lacks robust application of the PMC index model. Therefore, this study aims to collect relevant TCMRPs from 1985 to 2023 and utilize ROSTCM6 for analysis purposes while constructing an evaluation index system. Additionally, it will employ the PMC index model to analyze these collected policies in order to identify their strengths and weaknesses. The purpose of this study is to gain insights into the development process and current status of TCMRPs in China while providing valuable references for future formulation and improvement endeavors.

## 3 Research design

### 3.1 Data sources and samples selection

This paper focuses on the TCM registration policy promulgated by the Chinese government. Relevant policy documents were primarily retrieved from official websites such as The State Council, National Health Commission, National Medical Products Administration, National Administration of TCM, and other relevant government departments. Additionally, supplementary documents were searched for on platforms like Baidu, Zhiyun, Duxiu, and other related websites. We employed keywords such as “drug registration,” “Chinese medicine registration,” and “Chinese medicine approval.” Considering the continuous optimization of the TCMRPs, our search timeframe was set from January 1, 1985 to December 31, 2023. In this paper, duplicate and invalid files are screened based on the following principles: 1, Exclude laws and regulations not issued by national authorities. 2, Exclude documents that are not part of the drug registration policy. 3, Exclude policies unrelated to Chinese medicine. The policy documents primarily encompass legislation, regulations, strategic plans, outlines, and other pertinent provisions pertaining to pharmaceutical innovation in China. Therefore, this study initially collected 434 TCMDPs. To ensure the representativeness of the policies and the consistency of the evaluation process, a rigorous screening procedure was implemented. Based on predefined criteria, certain policy documents were excluded. Ultimately, a thorough analysis identified 165 policy texts that met the study requirements and aligned with the research objectives (Table 1 provides examples of the selected TCMDP).

**Table 1:** 28 of the 165 pharmaceutical innovation policies.

Code	Policy name	Publishing Entity	Date issued
P1	Drug Administration Law of the People’s Republic of China	The Standing Committee of the National People’s Congress	1985
P2	Measures for the Approval of New Drugs	The Ministry of Health of the People’s Republic of China(withdrawn)	1985
P3	Supplementary provisions and instructions on the issue of traditional Chinese Medicine in the Measures for the Approval of New Drugs	The Ministry of Health of the People’s Republic of China(withdrawn)	1987
P4	Some Supplementary Provisions on the Administration of New Drug Approval	The Ministry of Health of the People’s Republic of China(withdrawn)	1988
P5	Notice on Several Issues Concerning the Administration of Drug Approval	The Ministry of Health of the People’s Republic of China(withdrawn)	1992
P6	Guidelines for Pharmaceutical Research of New Traditional Chinese Medicine	The Ministry of Health of the People’s Republic of China(withdrawn)	1993
P7	Guiding Principles for Clinical Research of New Chinese Medicine	The Ministry of Health of the People’s Republic of China(withdrawn)	1993
P8	Guiding Principles for the Research of Traditional Chinese Medicine Injections	The Ministry of Health of the People’s Republic of China(withdrawn)	1993
P9	Guidelines for Research on New Toxicology of Traditional Chinese Medicine	The Ministry of Health of the People’s Republic of China(withdrawn)	1993
P10	Guide to Pharmacological Research on New Chinese Medicines	The Ministry of Health of the People’s Republic of China(withdrawn)	1993
P11	Measures for the Approval of New Drugs	The State Drug Administration (SDA)	1999
P12	Drug Administration Law of the People’s Republic of China	The Standing Committee of the National People’s Congress	2001
P13	Regulations for the Implementation of the Drug Administration Law	The State Council of the People’s Republic of China	2002
P14	Measures for the Administration of Drug Registration (Trial implementation)	The State Drug Administration (SDA)	2002
P15	Good Practice in Pharmaceutical Marketing (GAP)	The State Drug Administration (SDA)	2002
P16	Guidelines for Clinical Research of New Traditional Chinese Medicine	The State Drug Administration (SDA)	2002
P17	Supplementary Provisions on Drug Registration Administration	The State Food and Drug Administration (SFDA)	2003
P18	Good Clinical Practice (GCP)	The State Food and Drug Administration (SFDA)	2003
P19	Good Clinical Practice for Non-Clinical Research (GLP)	The State Food and Drug Administration (SFDA)	2003
P20	Measures for Drug Registration Administration	The State Food and Drug Administration (SFDA)	2005
P21	Measures for Drug Registration Administration	The State Food and Drug Administration (SFDA)	2007
P22	Supplementary Provisions on the Registration and Administration of Traditional Chinese Medicine	The State Food and Drug Administration (SFDA)	2008
P23	Opinions on Reforming the Review and Approval System of Drugs and Medical Devices	The China Food and Drug Administration (CFDA)	2015
P24	Law of the People’s Republic of China on Traditional Chinese Medicine	The China Food and Drug Administration (CFDA)	2017
P25	Regulations on Simplified Registration, Approval and Administration of Traditional Chinese Medicine Compound Preparations of Ancient Classical Prescriptions	The China Food and Drug Administration (CFDA)	2018
P26	Opinions on Promoting the Inheritance, Innovation and Development of Traditional Chinese Medicine	The State Council of the People’s Republic of China	2019
P27	Measures for Drug Registration Administration	The National Medical Products Administration (NMPA)	2020
P28	Special Regulations on the Registration and Administration of Traditional Chinese Medicine	The National Medical Products Administration (NMPA)	2023

### 3.2 Identification of the policy text features

We utilized the ROSTCM6 software to perform text mining and data processing on the collected policies, encompassing word segmentation, identification of high-frequency terms, and generation of network semantic maps. Non-meaningful words that appeared frequently were eliminated. The most pertinent and frequent terms were gathered for further analysis. The prevalence of certain terms in policy documents can indicate the government’s primary concerns. The semantic network diagram (see Figure 1) vividly illustrates the interrelation and significance of various terms in TCMRPs.

### 3.3 Construction of the PMC index model

**Figure 2:**
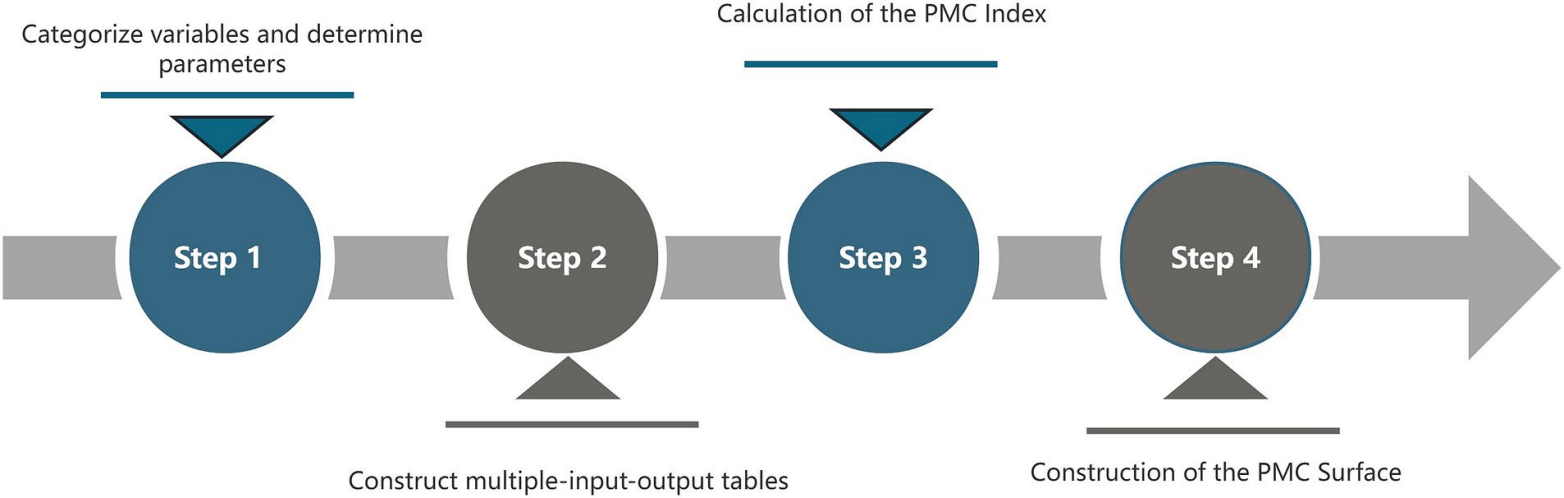
Diagram of the steps to build the PMC model.

The PMC index model is a scientific and quantitative method for policy evaluation, proposed by Estrada (48) and derived from the Omnia Mobilis hypothesis. This hypothesis posits that all elements are in constant motion and interconnected, emphasizing the importance of considering seemingly unrelated variables without limitations on their number or weight. By employing a comprehensive selection of variables, the PMC index model assesses the merits and drawbacks of each strategy while evaluating its consistency across multiple dimensions. Comprising four main steps, this model offers an enhanced approach to policy analysis (see Figure 2).

#### 3.3.1 Variable classification and parameter identification

The foundation of policy comprehensive evaluation relies on a rational classification of variables and a scientific identification of parameters. Based on the analysis results obtained from ROSTCM6 software and considering the specific characteristics of TCMRPs, we have established nine primary variables: the nature of the policy (X1), the duration of the policy (X2), the issuing institution (X3), the policy object (X4), the policy function (X5), the policy content (X6), the policy method (X7), the incentive method (X8), and the policy science (X9). By extracting key variables and defining sub-variables through relevant literature and policies, we have identified 41 sub-variables as shown in Table 2. Parameters are assigned after proper classification. A binary approach is employed to assign equal weights to all sub-variables, if a particular policy aligns with a given sub-variable, its parameter is set to 1, otherwise, it is set to 0.

**Table 2:** Pharmaceutical Innovation Policy Evaluation Framework (Selected Indicators).

Primary variable	Sub-variables	Evaluation criteria	Reference
Nature of policy (X1)	Forecast (X1-1)	Determine if the policy reflects the forecast	(48)
	Guidance (X1-2)	Determine if the policy reflects guidance	
	Description (X1-3)	Determine if the policy reflects the description	
	Regulation (X1-4)	Determine if the policy reflects regulation	
	Plan (X1-5)	Determine if the policy reflects the plan	
	Support (X1-6)	Determine if the policy reflects support	
Policy timeliness (X2)	Long term (3–5 years) (X2-1)	Determine if the policy is a long-term policy	(49)
	Short term (1–3 years) (X2-2)	Determine if the policy is a short-term policy	
	Within one year (X2-3)	Determine if the policy is a one-year limitation policy	
Issuing Institution (X3)	Country (X3-1)	Determine if the policy is issued by a state agency	(50)
	Autonomous Regions and municipalities (X3-2)	Determine whether the policy is issued by an autonomous region and municipality	
	Administrative departments (X3-3)	Determine if the policy is issued by each executive branch	
	Other (X3-4)	Determine if the policy is issued for other agencies	
Object of Policy (X4)	Authority (X4-1)	Determine if the target of the policy is an authority	(51)
	Business (X4-2)	Determine if the policy is aimed at a business	
	Hospital (X4-3)	Determine if the policy is targeted to a hospital	
	Laboratory (X4-4)	Determine if the subject of the policy is a laboratory	
Policy Function (X5)	Guiding Development (X5-1)	Determine if the policy function has guided development	(52)
	Prescriptive Standards (X5-2)	Determine if the policy function has a prescribed standard	
	Optimization Procedure (X5-3)	Determine if the policy function has an optimization procedure	
	Encourage Innovation (X5-4)	Determine whether the policy function is encouraging innovation	
	Optimize product structure (X5-5)	Determine if the policy function has an optimized product structure	
	Perfect the system (X5-6)	Determine if the policy function has a perfect system	
Policy Content (X6)	Technical Guidance (X6-1)	Determine if the policy content has technical guidance	(53)
	Prioritizing resources (X6-2)	Determine if the policy content has a priority allocation resource	
	Shorten review time limit (X6-3)	Determine if the policy content has a shortened review time limit	
	The application requirements (X6-4)	Determine if the policy content is eligible for application	
	Application Path (X6-5)	Determine if the policy content has an application path	
	Registration Verification and Inspection (X6-6)	Determine if the policy content has registration verification and inspection	
Policy Approach (X7)	Coercive (X7-1)	Determine if the policy approach is coercive	(15)
	Service Type (X7-2)	Determine if the policy approach is service-oriented	
	Incentive-based (X7-3)	Determine if the policy approach is incentive	
Incentive Method (X8)	Program Simplification (X8-1)	Determine if the policy incentive method has procedural simplification	(15)
	Enrollment Subsidy (X8-2)	Determine if the policy incentive method has a enrollment subsidy	
	Intellectual Property Protection (X8-3)	Determine whether the policy incentive method has intellectual property protection	
	Regulation and Evaluation (X8-4)	Determine whether the policy incentive method has regulation and evaluation	
Scientificity of policy (X9)	Well-founded (X9-1)	Determine whether the policy is scientifically sound	(54)
	Detailed content (X9-2)	Determine whether the policy is scientific and detailed	
	Detail Measures (X9-3)	Determine whether the policy is scientific and refined measures	
	Clear division of Labor (X9-4)	Determine whether the scientific nature of the policy has a clear division of labor	
	Clear responsibilities and rights (X9-5)	Determine whether the scientific nature of the policy is clear in terms of responsibilities and rights	

#### 3.3.2 Building a multi-input–output table

The PMC index model for TCMRPs requires the construction of a multi-input–output table, which utilizes an analysis framework capable of storing data and evaluating single variables across multiple dimensions. This study employs a multi-input–output table consisting of 9 principal variables and 41 sub-variables. The main variables are independent of each other, with no specific ranking order, while the sub-variables serve as refined measures of the primary variables. Separate analyses are conducted to determine whether sub-variables are involved in the same policy. Preliminary findings indicate that, except for a few variables, the evaluation results generally align with other variables. Further analysis and discussion focus on both internal and scientific policies to obtain scientifically sound secondary variables. After parameter identification, a multi-output input table comprising 165 TCMRPs is established.

#### 3.3.3 Measurement of the PMC index

According to the PMC index model proposed by Estrada (11), the calculation method is as follows: Firstly, assign values to each secondary variable (Equation 1). Then, calculate the values of each level variable of the TCM registration policy using Equation 2. Finally, all first-level variables will be added and calculated according to Equation 3 and Equation 4 to obtain the PMC index value for evaluating the policy. Here, Xi represents the i-th principal variable where i ranges from 1 to 9. Xij represents the i-th subvariable where j ranges from 1 to n. Follow these steps to calculate the PMC index for 165 TCM registration policies. The PMC index is used to evaluate the comprehensiveness and consistency of policies within our policy evaluation system that selects nine first-order variables, resulting in a range between 0 and 9 for the PMC index value indicating its acceptability level. Based on existing research findings, we have divided the PMC index into four evaluation levels: A perfect policy falls within a range of 8.00–9.00, excellent consistency is indicated by a range between 6.00 and <8 0.00, an acceptable range lies between 4 0.00 and <6 0.000, while a value less than 4 indicates poor acceptability and inadequate policy quality (Table 3).


(1) X∼N≤[0,1]

(2) X={XR:[0,1]}

(3) $$X_{1}(\sum_{j=1}^{n}\frac{X_{\mathrm{ij}}}{T(X_{\mathrm{ij}})})$$

(4) $$\mathrm{PMC}=[\begin{array}{l} X_{1}(\sum_{j=1}^{6}\frac{X_{1j}}{6})+X_{2}(\sum_{j=1}^{6}\frac{X_{2j}}{6})\\ +X_{3}(\sum_{j=1}^{4}\frac{X_{3j}}{4})+X_{4}(\sum_{j=1}^{4}\frac{X_{4j}}{4})\\ +X_{5}(\sum_{j=1}^{3}\frac{X_{5j}}{3})+X_{6}(\sum_{j=1}^{3}\frac{X_{6j}}{3})\\ +X_{7}(\sum_{j=1}^{5}\frac{X_{7j}}{5})+X_{8}(\sum_{j=1}^{5}\frac{X_{8j}}{5})\\ +X_{9}(\sum_{j=1}^{4}\frac{X_{9j}}{4}) \end{array}]$$

**Table 3:** Evaluation criteria for policy based on the PMC-Index.

PMC-Index	0 ≤ PMC<4	4 ≤ PMC<6	6 ≤ PMC<8	8 ≤ PMC<9
Evaluation	Bad	Good	Excellent	Perfect

#### 3.3.4 Construction of the PMC-surface

The drawing of a PMC surface map provides a more intuitive representation of the emphasis placed on each variable, allowing for a visualization of the policy’s advantages and disadvantages across multiple dimensions. In this study, a 3 × 3 matrix is utilized to incorporate 9 first-order variables and create a 3D PMC surface map. Equation 5 illustrates the calculation method employed for generating the PMC surface. The horizontal axis (X) and vertical axis (Y) are used to establish two-dimensional coordinates for each first-order variable, determining their specific positions within the PMC surface diagram. Consequently, both the concavity and color depth of the PMC-Surface directly reflect the strengths and weaknesses associated with each policy. MATLAB software is employed to generate the PMC-Surface diagram.


(5) $$\mathrm{PMC}-\text{surface}=[\begin{array}{ccc} X_{1} & X_{2} & X_{3}\\ X_{4} & X_{5} & X_{6}\\ X_{7} & X_{8} & X_{9} \end{array}]$$

## 4 Results and discussion

### 4.1 Analysis of high-frequency words

**Figure 3:**
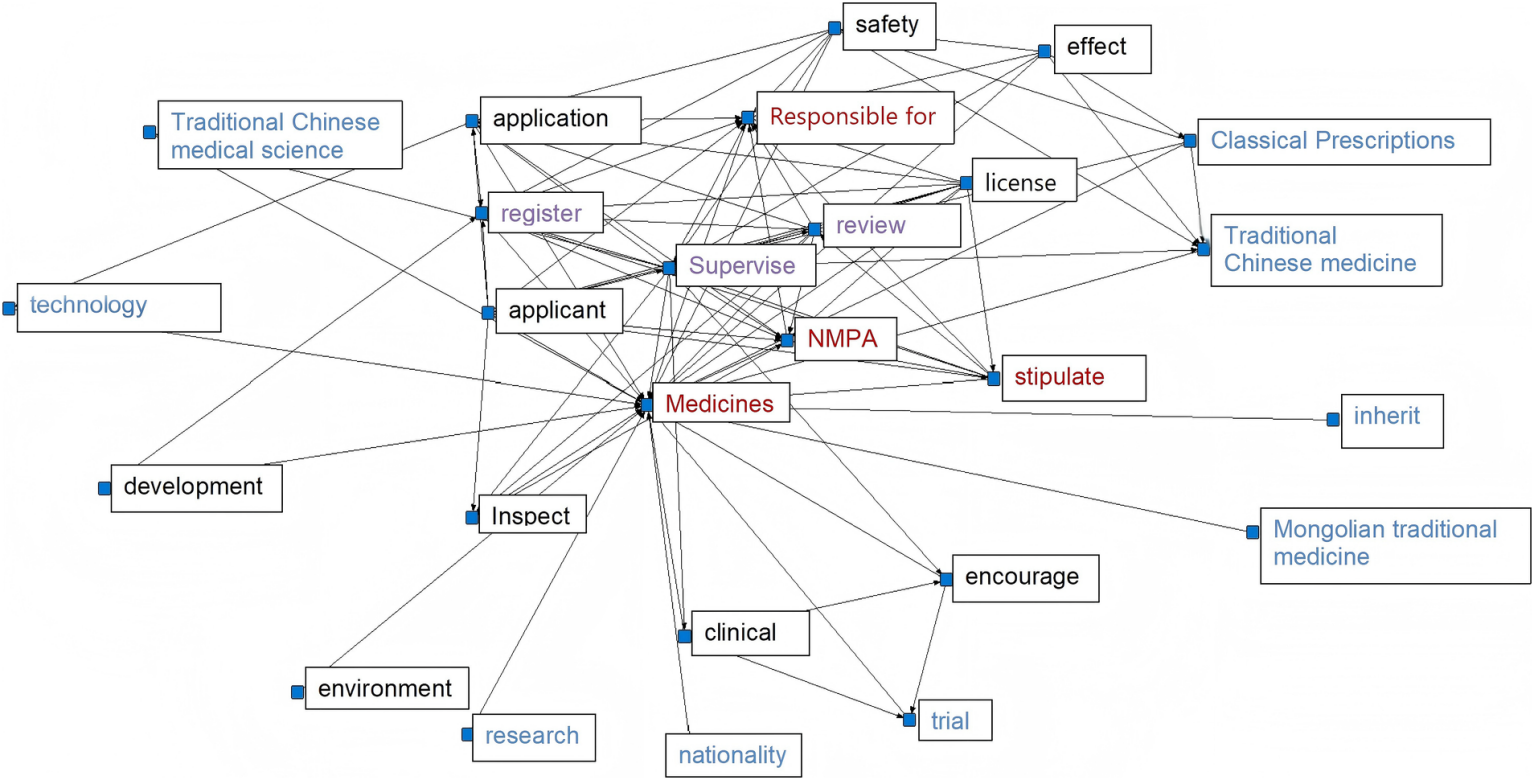
2008 TCM Registration Policy Network Semantic Graph.

In 2008, the lack of specific policies for the registration of TCM resulted in most available policies being related to general drug regulations. The registration of new TCM falls under the broader framework of drug registration. A high-frequency word analysis of the drug registration policy indicates that the National Medical Products Administration (NMPA) has been explicitly designated as the primary authority responsible for drug registration, with its duties including the review, approval, and oversight of drug registration procedures (Figure 3). Analysis of high-frequency words in various policies indicates certain similarities, with terms like “clinical,” “regulatory,” “national,” “inspection,” and “review” appearing consistently across almost all policies. This highlights a significant emphasis placed on new drug registration, where strict procedures and national supervision play pivotal roles. Additionally, high-ranking terms such as “TCM,” “technology,” “classic famous prescription,” and “encourage” suggest active promotion by national policies toward innovative development within TCM culture, technical advancements in TCM practices, resource expansion for TCM, and enhancement of its innovation system.

The high-frequency terms found in the Supplementary Provisions on the Registration and Administration of TCM, the Law of the People’s Republic of China on TCM, and the Regulations on Simplified Registration and Approval of TCM Compound Preparations all involve “research,” “technology,” “Chinese medicine,” and other related terms. This indicates that TCM is closely linked to research and technology, with TCM serving as its foundation for development. Technology is a necessary product for improving inheritance and development within TCM, therefore, investment in research is required. Furthermore, “clinical trials” appear frequently among these high-frequency terms which suggests that new drugs derived from TCM must undergo extensive clinical testing before being approved for market use. Although this may slow down registration processes for new drugs derived from TCM, it ensures their safety once they are available to consumers. High-frequency words located at the edge of network semantic graphs should not be overlooked either - among them lies one particularly special term: “ancient classic prescription.” While ranking relatively low compared to other high-frequency words mentioned earlier, ancient classic prescriptions represent an invaluable treasure trove of knowledge regarding ancient traditions within Chinese medicinal culture. The policy of Simplifying the Registration and Approval Management Regulations of Ancient Classic Famous Chinese Medicine Compound Preparations clearly stipulates that all new Chinese medicine belonging to ancient classic famous Chinese medicine can streamline certain approval procedures, thereby demonstrating the government’s strong commitment to the inheritance and innovative development of TCM. Additionally, high-frequency terms such as “Mongolian medicine,” “environment,” “inheritance,” and “culture” on the periphery indicate a significant emphasis placed by the Chinese government on ethnic medicine. Ethnic medicine serves as a cultural reflection, thus safeguarding its inheritance and protection is tantamount to preserving our culture. Furthermore, it is essential to prioritize environmental conservation while actively promoting advancements in the new drug registration industry—a testament to China’s dedication toward environmental preservation at a global scale.

### 4.2 Index analysis and comparison of medicine innovation policy

#### 4.2.1 Index analysis of TCMRPs

According to the aforementioned evaluation system and criteria, the PMC index is calculated to determine the level of the new drug registration policy for TCM, as illustrated in the table provided (Table 4). The average PMC index value of 165 comprehensive evaluation indicators for new drug registration policies was found to be 5.858, indicating an excellent overall consistency among these comprehensive evaluation indicators. The specific count reveals the existence of 39 policies that are deemed excellent, 119 policies that are considered acceptable, 7 policies with poor consistency, and no perfectly consistent policies. These policies were primarily issued by authorities such as the former Ministry of Health (now revoked), the State Food and Drug Administration, the National Health Commission, and the State Administration of TCM. This demonstrates significant attention given by the Chinese government toward TCM. Moreover, it highlights their determination to rigorously approve new TCMRPs in order to promote both inheritance and innovation within TCM development.

**Table 4:** A comprehensive review of 165 Traditional Chinese Medicine Innovation Policies.

Policy name	X1	X2	X3	X4	X5	X6	X7	X8	X9	PMC index
Drug Administration Law of the People’s Republic of China, 1985	0.67	1	0.25	1	0.5	0.5	0.67	0.5	0.8	5.89
New Drug (Traditional Chinese Medicine) Application Materials Project, 1985	0.33	0.67	0.25	0.5	0.33	0.33	0.67	0.25	0.6	3.93
Regulations on the Protection of New Drugs and Technology Transfer, 1985	0.5	0.67	0.25	0.5	0.33	0.33	0.67	0.25	0.4	3.9
Measures for the Approval of New Drugs 1985	0.5	1	0.25	1	0.33	0.67	0.67	0.5	0.8	5.72
Supplementary Provisions and Instructions on the Issue of Traditional Chinese Medicine in the Measures for Approval of New Drugs 1987	0.83	0.67	0.25	1	0.83	0.5	0.67	0.25	0.4	5.4
Some Supplementary Provisions on the Approval and Administration of New Drugs 1988	0.5	0.67	0.25	1	0.5	0.67	0.67	0.5	0.6	5.36
The Revision and Supplementary Provisions of the Measures for the Examination and Approval of New Drugs Concerning Traditional Chinese Medicine 1992	0.5	0.67	0.25	1	0.5	0.33	0.67	0.5	0.6	5.02
Notice on Several Issues Concerning the Administration of Drug Approval, 1992	0.83	1	0.25	1	0.83	0.67	1	0.5	0.8	6.88
Supplementary Notice on the Approval of Confidential New Chinese Medicine Varieties, 1993	0.67	1	0.25	1	0.5	0.67	1	0.5	0.6	6.19
Guiding Principles for Clinical Research of New Traditional Chinese Medicine	0.67	1	0.25	1	0.5	0.67	0.67	0.5	0.6	5.86
“Guidelines for Research on New Drugs of Traditional Chinese Medicine” Pharmacy, Pharmacology, and Toxicology	0.67	1	0.25	1	0.5	0.67	0.67	0.5	0.6	5.86
Guiding Principles for the Development of Traditional Chinese Medicine Injections	0.67	1	0.25	1	0.5	0.67	0.67	0.5	0.6	5.86
Guidelines for Pharmaceutical Research of New Traditional Chinese Medicine	0.67	1	0.25	1	0.67	0.5	0.67	0.5	0.6	5.86
Guiding Principles for Clinical Research of New Chinese Medicine	0.5	1	0.25	1	0.5	0.67	0.67	0.5	0.4	5.49
Guiding Principles for the Research of Traditional Chinese Medicine Injections	0.5	1	0.25	1	0.5	0.67	0.67	0.5	0.4	5.49
Guidelines for Research on New Toxicology of Traditional Chinese Medicine	0.5	1	0.25	1	0.5	0.67	0.67	0.5	0.4	5.49
Regulations on the Protection of New Drugs and Technology Transfer 1999	0.67	1	0.25	1	0.5	0.5	0.67	0.5	0.6	5.69
Annex 2: Application Materials for New Drugs (Traditional Chinese Medicine) 1999	0.33	0.67	0.25	0.5	0.33	0.33	0.67	0.25	0.6	3.93
Guidelines for Pharmacological Research on New Chinese Medicine	0.5	1	0.25	1	0.5	0.67	0.67	0.5	0.4	5.49
Measures for the Approval of New Drugs, 1999	0.67	1	0.25	1	0.5	0.67	0.67	0.5	0.8	6.06
Good Manufacturing Practice (revised in 1998) 1999	0.67	0.67	0.25	1	0.5	0.33	0.67	0.5	0.6	5.19
Standard for Quality Control of Drug Trade (Order No. 20 of the Bureau) 2000	0.5	0.67	0.25	1	0.5	0.33	0.67	0.5	0.6	5.02
Drug Administration Law of the People’s Republic of China, 2001	0.67	1	0.25	1	0.67	0.67	0.67	0.5	0.8	6.23
Annex 1: Classification and Application Requirements for Registration of Traditional Chinese Medicine and natural Medicine 2002	0.33	0.67	0.25	0.5	0.33	0.33	0.67	0.25	0.6	3.93
Regulations for the Implementation of the Drug Administration Law, 2002	0.83	1	0.25	1	0.67	0.67	0.67	0.5	0.6	6.19
Measures for the Administration of Drug Registration (for trial implementation)2002	0.67	0.67	0.25	1	0.5	0.5	0.33	0.5	0.4	4.82
Good Practice for Drug Marketing (GAP)2002	0.67	0.67	0.25	1	0.5	0.5	0.67	0.5	0.6	5.36
Guidelines for Clinical Research of New Traditional Chinese Medicine, 2002	0.5	1	0.25	1	0.5	0.67	0.67	0.5	0.4	5.49
Supplementary Provisions on the Administration of Drug Registration 2003	0.67	0.67	0.25	1	0.67	0.67	0.67	0.5	0.6	5.7
Good Clinical Practice (GCP)2003	0.67	0.67	0.25	1	0.5	0.5	0.67	0.5	0.6	5.36
Good Clinical Practice for Non-Clinical Research (GLP) 2003	0.67	0.67	0.25	1	0.5	0.5	0.67	0.5	0.6	5.36
Measures for the Supervision and Administration of Drug Production in 2004	0.67	0.67	0.25	1	0.5	0.5	0.67	0.5	0.6	5.36
Measures for the Administration of Packaging Materials and Containers for Direct Contact with Pharmaceuticals 2004	0.33	0.67	0.25	0.5	0.5	0.5	0.33	0.5	0.6	4.18
Annex 1: Classification and Application Requirements for Registration of Traditional Chinese Medicine and natural Medicine 2005	0.33	0.67	0.25	0.5	0.33	0.33	0.67	0.25	0.6	3.93
Procedures and requirements for on-site inspection and sampling of drug registration (trial implementation) 2005	0.5	0.67	0.25	0.5	0.5	0.33	0.67	0.5	0.6	4.52
Special Procedures for Drug Approval by the State Food and Drug Administration, 2005	0.67	0.67	0.25	1	0.67	0.67	0.67	0.5	0.6	5.7
Measures for Drug Registration Administration, 2005	0.67	0.67	0.25	1	0.5	0.5	0.67	0.5	0.4	5.16
Technical guidelines for research on extraction and purification of traditional Chinese medicine and natural drugs	0.67	1	0.25	1	0.5	0.67	0.67	0.5	0.6	5.86
Technical guidelines for research on traditional Chinese medicine and natural medicine preparations	0.67	1	0.25	1	0.5	0.67	0.67	0.5	0.6	5.86
Technical guidelines for pre-treatment of raw materials of traditional Chinese medicine and natural medicine	0.67	1	0.25	1	0.5	0.67	0.67	0.5	0.6	5.86
Technical guidelines for pilot study of traditional Chinese medicine and natural drugs	0.67	1	0.25	1	0.5	0.67	0.67	0.5	0.6	5.86
Regulations on the Administration of Drug Instructions and Labels 2006	0.5	1	0.25	0.5	0.5	0.67	0.67	0.75	0.8	5.64
Guiding Principles for writing prescription drug instructions of traditional Chinese medicine and natural medicine	0.67	1	0.25	1	0.5	0.67	0.67	0.5	0.6	5.86
Requirements for writing the contents of prescription instructions of traditional Chinese medicine and natural medicine	0.67	1	0.25	1	0.5	0.67	0.67	0.5	0.6	5.86
Technical Guidelines for Stability Research of Traditional Chinese Medicine and natural Drugs	0.67	1	0.25	1	0.5	0.67	0.67	0.5	0.6	5.86
Measures for the Supervision and Administration of Drug Circulation (Order No. 26 of the Bureau) 2007	0.67	1	0.25	0.5	0.67	0.5	0.67	0.5	0.6	5.36
Measures for the Administration of Drug Registration 2007	0.67	0.67	0.25	1	0.67	0.67	0.67	0.5	0.4	5.5
Standards for Review and Publication of Drug Advertisements 2007	0.67	1	0.25	0.5	0.67	0.5	0.67	0.75	0.6	5.61
Technical guidelines for the format and content of review materials for traditional Chinese medicine and natural medicine -- Summary and evaluation of the main research results	0.67	1	0.25	1	0.5	0.67	0.67	0.5	0.6	5.86
Technical guidelines for the format and content of review materials of traditional Chinese medicine and natural medicine -- Clinical research review	0.67	1	0.25	1	0.5	0.67	0.67	0.5	0.6	5.86
Technical guidelines for the format and content of review materials on traditional Chinese medicine and natural medicine: a review of pharmacological and toxicological studies	0.67	1	0.25	1	0.5	0.67	0.67	0.5	0.6	5.86
Technical guidelines for the format and content of review materials for traditional Chinese medicine and natural medicine: a review of pharmaceutical research materials	0.67	1	0.25	1	0.5	0.67	0.67	0.5	0.6	5.86
Annex 1: Classification and application requirements for registration of Traditional Chinese Medicine and natural Medicine 2007	0.33	0.67	0.25	0.5	0.33	0.33	0.67	0.25	0.6	3.93
Technical guidelines for long-term toxicity study of traditional Chinese medicine and natural drugs	0.67	1	0.25	1	0.5	0.67	0.67	0.5	0.6	5.86
Technical guidelines for the study of acute toxicity of traditional Chinese medicine and natural drugs	0.67	1	0.25	1	0.5	0.67	0.67	0.5	0.6	5.86
Medical theory and principles of literature writing for clinical research of traditional Chinese medicine and natural drugs	0.67	1	0.25	1	0.5	0.67	0.67	0.5	0.6	5.86
Technical guidelines for research on local irritation and hemolysis of traditional Chinese medicine and natural medicine	0.67	1	0.25	1	0.5	0.67	0.67	0.5	0.6	5.86
Principles for writing clinical trial reports of traditional Chinese medicine and natural drugs	0.67	1	0.25	1	0.5	0.67	0.67	0.5	0.6	5.86
Technical guidelines for general pharmacological research of traditional Chinese medicine and natural drugs	0.67	1	0.25	1	0.5	0.67	0.67	0.5	0.6	5.86
Technical guidelines for research on immunotoxicity (allergy, photoallergy) of traditional Chinese medicine and natural drugs	0.67	1	0.25	1	0.5	0.67	0.67	0.5	0.6	5.86
Principles for writing drug instructions of traditional Chinese medicine and natural medicine	0.67	1	0.25	1	0.5	0.67	0.67	0.5	0.6	5.86
Technical guidelines for drug genotoxicity research	0.67	1	0.25	1	0.5	0.67	0.67	0.5	0.6	5.86
Technical guidelines for clinically independent drug studies	0.67	1	0.25	1	0.5	0.67	0.67	0.5	0.6	5.86
Basic technical requirements for injections of traditional Chinese medicine and natural drugs	0.67	1	0.25	1	0.5	0.67	0.67	0.5	0.6	5.86
Supplementary Provisions on the Registration and Administration of Traditional Chinese Medicine, 2008	0.83	1	0.25	1	0.67	0.67	0.67	0.5	0.6	6.19
Regulations on the Administration of On-site Inspection of Drug Registration in 2008	0.5	1	0.25	1	0.67	0.67	0.67	0.5	0.6	5.86
Special Regulations on the Registration and Administration of Traditional Chinese Medicine in 2008	0.83	1	0.25	1	0.67	0.67	0.67	0.5	0.6	6.19
Determination criteria and treatment principles for ambiguous quality standards of traditional Chinese medicine	0.67	1	0.25	1	0.5	0.67	0.67	0.5	0.6	5.86
Principles for the treatment of Chinese medicinal species containing endangered medicinal materials	0.67	1	0.25	1	0.5	0.67	0.67	0.5	0.6	5.86
Principles for dealing with problems related to the process of traditional Chinese medicine	0.67	1	0.25	1	0.5	0.67	0.67	0.5	0.6	5.86
Principles for the treatment of toxic medicinal materials and other Chinese medicine varieties with safety problems	0.67	1	0.25	1	0.5	0.67	0.67	0.5	0.6	5.86
Principles for dealing with problems related to TCM external preparations	0.67	1	0.25	1	0.5	0.67	0.67	0.5	0.6	5.86
Principles of dealing with problems related to quality control research of traditional Chinese medicine	0.67	1	0.25	1	0.5	0.67	0.67	0.5	0.6	5.86
Regulations on the Administration of Special Approval for New Drug Registration 2009	0.83	1	0.25	1	0.67	0.67	0.67	0.5	0.6	6.19
Technical guidelines for the necessity of carcinogenicity testing of drugs	0.67	1	0.25	1	0.5	0.67	0.67	0.5	0.6	5.86
Technical requirements for rational selection of dosage forms of modified traditional Chinese medicine	0.67	1	0.25	1	0.5	0.67	0.67	0.5	0.6	5.86
Regulations for the Administration of Pharmaceutical Production, Decree No. 79 of the Ministry of Health, 2011	0.5	0.67	0.25	1	0.5	0.5	0.67	0.5	0.6	5.19
Regulation and arrangement of drug registration and application materials	0.5	1	0.25	1	0.5	0.5	0.67	0.5	0.6	5.52
Technical guidelines for Research on Alteration of Marketed Traditional Chinese Medicine (1)	0.67	1	0.25	1	0.5	0.67	0.67	0.5	0.6	5.86
Guiding principles for the management of Phase I drug clinical trials (Trial)	0.33	0.67	0.25	1	0.5	0.5	0.33	0.5	0.6	4.68
Guiding Principles for Drug Interaction Research 2012	0.5	1	0.25	1	0.5	0.5	0.67	0.25	0.6	5.27
Opinions of the State Food and Drug Administration on Deepening the Reform of Drug evaluation and Approval and Further Encouraging Drug Innovation, 2013	0.83	1	0.25	1	0.67	0.67	0.67	0.5	0.6	6.19
Traditional Chinese Medicine Law of the People’s Republic of China, 2013	0.67	1	0.25	1	0.83	0.83	0.67	0.75	0.6	6.6
Standard for Quality Control of Drug Distribution (Decree No. 90 of the Ministry of Health), 2013	0.67	0.67	0.25	1	0.5	0.67	0.67	0.5	0.8	5.73
Regulations on Simplified Registration and Approval Administration of Compound Preparations of Ancient Famous Prescriptions of Traditional Chinese Medicine, 2013	0.67	0.67	0.25	1	0.67	0.67	0.67	0.5	0.6	5.7
Opinions on Promoting the Inheritance, Innovation and Development of Traditional Chinese Medicine, 2013	0.83	1	0.25	1	0.67	0.67	0.67	0.67	0.6	6.36
Technical requirements for New natural medicine Research 2013	0.67	1	0.25	1	0.5	0.67	0.67	0.5	0.6	5.86
Technical guidelines for research on modified dosage forms of traditional Chinese medicine and natural medicines 2014	0.67	1	0.25	1	0.5	0.67	0.67	0.5	0.6	5.86
The potential effect of drugs on QT interval prolongation is not a technical guideline for clinical research	0.67	1	0.25	1	0.5	0.67	0.67	0.5	0.6	5.86
Technical guidelines for pharmacological research on drug safety	0.67	1	0.25	1	0.5	0.67	0.67	0.5	0.6	5.86
Technical guidelines for drug irritant, allergic, and hemolytic research	0.67	1	0.25	1	0.5	0.67	0.67	0.5	0.6	5.86
Technical guidelines for single-dose drug toxicity studies	0.67	1	0.25	1	0.5	0.67	0.67	0.5	0.6	5.86
Technical guidelines for drug toxicokinetics studies	0.67	1	0.25	1	0.5	0.67	0.67	0.5	0.6	5.86
Technical guidelines for non-clinical pharmacokinetic studies of drugs	0.67	1	0.25	1	0.5	0.67	0.67	0.5	0.6	5.86
Technical guidelines for toxicity studies of repeated drug administration	0.67	1	0.25	1	0.5	0.67	0.67	0.5	0.6	5.86
Opinions on Reforming the Review and Approval System for Drugs and Medical Devices, 2015	0.67	1	0.25	1	0.67	0.67	0.67	1	0.4	6.33
Good Practice for Drug Distribution 2015	0.67	0.67	0.25	1	0.5	0.67	0.67	0.5	0.8	5.73
General principles of clinical research on new traditional Chinese medicine	0.67	1	0.25	1	0.5	0.67	0.67	0.75	0.6	6.11
Good Practice for Drug Distribution 2016	0.67	1	0.25	1	0.67	0.67	0.67	1	0.6	6.53
Law of the People’s Republic of China on Traditional Chinese Medicine, 2017	0.83	1	0.25	1	0.83	0.83	0.67	0.75	0.6	6.76
Good Clinical Practice for Non-Clinical Research, 2017	0.67	0.67	0.25	1	0.67	0.67	0.67	0.5	0.6	5.7
Opinions on Deepening the Reform of the Review and Approval System and Encouraging Innovation of Drugs and Medical Devices, 2017	0.83	1	0.25	1	0.67	0.67	0.67	1	0.6	6.69
Measures for the Supervision and Administration of Drug Production 2017	0.67	1	0.25	1	0.5	0.67	0.67	0.75	0.6	6.11
Guidelines for the Review of Registration, Approval and Acceptance of Traditional Chinese Medicine and Natural Medicine (Trial)	0.83	1	0.25	1	0.67	0.67	0.67	0.67	0.6	6.36
Technical guidelines for research on the change of production process of marketed traditional Chinese medicine	0.67	1	0.25	1	0.5	0.67	0.67	0.5	0.6	5.86
Technical guidelines for naming generic names of Chinese patent medicines	0.67	1	0.25	1	0.5	0.67	0.67	0.5	0.6	5.86
Specification of Chinese patent medicine expresses technical guiding principles	0.67	1	0.25	1	0.5	0.67	0.67	0.5	0.6	5.86
The Regulations on Simplified Registration, Approval and Administration of Traditional Chinese Medicine Compound Preparations of Ancient Famous Prescriptions, 2018	0.67	1	0.25	1	0.67	0.67	0.67	1	0.8	6.73
Communication Measures for Drug Research and Development and Technical Evaluation 2018	0.83	1	0.25	1	0.83	0.67	0.67	0.75	0.6	6.6
Technical guidelines for clinical research of new Chinese medicine for syndromes	0.67	1	0.25	1	0.67	0.67	0.67	0.75	0.6	6.28
Technical guidelines for drug genotoxicity research	0.67	1	0.25	1	0.67	0.67	0.67	0.75	0.6	6.28
Guidelines for clinical evaluation of liver injury induced by traditional Chinese medicine	0.67	1	0.25	1	0.67	0.67	0.67	0.75	0.6	6.28
Opinions on Promoting the Inheritance and Innovation of Traditional Chinese Medicine (TCM), 2019	0.67	1	0.25	1	0.83	0.83	0.67	1	0.8	7.05
Drug Administration Law of the People’s Republic of China, 2019	0.83	1	0.25	1	0.83	0.83	0.67	0.75	0.8	6.96
Regulations for the Implementation of the Drug Administration Law of the People’s Republic of China, 2019	0.83	1	0.25	1	0.83	0.83	0.67	0.75	0.8	6.96
Guidelines for the Review of Registration Acceptance of Traditional Chinese Medicine (Draft), 2020	0.83	1	0.25	1	0.67	0.67	0.67	0.75	0.8	6.64
Measures for the Administration of Drug Registration 2020	0.83	1	0.25	1	1	0.83	1	0.75	1	7.66
Annex 1: Classification of Chinese Medicine Registration and Application information requirements 2020	0.33	0.67	0.25	0.5	0.33	0.33	0.67	0.25	0.6	3.93
The 14th Five-Year Plan for National Drug Safety and High-quality Development, 2020	0.83	1	0.25	1	0.67	0.83	0.67	0.75	0.8	6.8
Implementation Opinions of the State Food and Drug Administration on Promoting the Inheritance, Innovation and Development of Traditional Chinese Medicine, 2020	0.83	1	0.25	1	0.67	0.83	0.67	0.75	0.8	6.8
Good Clinical Practice 2020	0.83	1	0.25	1	0.67	0.67	0.67	0.75	0.6	6.44
Technical guidelines for the Study of quality standards for New Traditional Chinese Medicine (Draft)	0.67	1	0.25	1	0.5	0.67	0.67	0.5	0.6	5.86
Guiding principles for drug development and evaluation supported by real-world evidence (trial)	0.67	1	0.25	1	0.5	0.67	0.67	0.5	0.6	5.86
Guidelines on Data Submission for Drug Clinical Trials (Trial)	0.67	1	0.25	1	0.5	0.67	0.67	0.5	0.6	5.86
Guiding Principles for the Noninferiority Design of Drug Clinical Trials	0.67	1	0.25	1	0.5	0.67	0.67	0.5	0.6	5.86
Guidelines for Drug Clinical Trial Data Monitoring Committee (Trial)	0.67	1	0.25	1	0.5	0.67	0.67	0.5	0.6	5.86
Technical guidelines for research on quality standards for New Traditional Chinese Medicine (Trial)	0.67	1	0.25	1	0.5	0.67	0.67	0.5	0.6	5.86
Technical guidelines for Research on quality control of Medicinal materials for New Traditional Chinese Medicine (Trial)	0.67	1	0.25	1	0.5	0.67	0.67	0.5	0.6	5.86
Technical guidelines for pharmaceutical research at different stages of New Drug Research in Traditional Chinese Medicine (Trial)	0.67	1	0.25	1	0.5	0.67	0.67	0.5	0.6	5.86
Technical guidelines for Homogenization Research of Traditional Chinese Medicine (Trial)	0.67	1	0.25	1	0.5	0.67	0.67	0.5	0.6	5.86
Pharmaceutical data requirements for communication meetings in the process of new Chinese medicine research (trial)	0.67	1	0.25	1	0.5	0.67	0.67	0.5	0.6	5.86
Technical guidelines for Marketing of Drugs with conditional approval (Trial)	0.67	1	0.25	1	0.5	0.67	0.67	0.5	0.6	5.86
Technical guidelines for Research on the Production Process of Compound Preparations of Traditional Chinese Medicine (Trial)	0.67	1	0.25	1	0.5	0.67	0.67	0.5	0.6	5.86
Technical guidelines for Research on Biological effect Detection of Traditional Chinese Medicine (Trial)	0.67	1	0.25	1	0.5	0.67	0.67	0.5	0.6	5.86
Guidelines for subgroup analysis in drug clinical trials (trial)	0.67	1	0.25	1	0.5	0.67	0.67	0.5	0.6	5.86
Guidelines for covariate adjustment in drug clinical trials	0.67	1	0.25	1	0.5	0.67	0.67	0.5	0.6	5.86
Guidelines on multiplicity in drug clinical trials (for trial implementation)	0.67	1	0.25	1	0.5	0.67	0.67	0.5	0.6	5.86
“14th Five-Year Plan for Drug Safety and High-quality Development” 2021	0.83	1	0.25	1	1	0.83	1	1	0.8	7.71
Technical guidelines for Quality Research of New Traditional Chinese Medicine (Trial)	0.67	1	0.25	1	0.5	0.67	0.67	0.5	0.6	5.86
Technical guidelines for the Study of Drug Interactions (Trial)	0.67	1	0.25	1	0.5	0.67	0.67	0.5	0.6	5.86
Guidelines for the adaptive design of drug clinical trials (trial)	0.67	1	0.25	1	0.5	0.67	0.67	0.5	0.6	5.86
Technical guidelines for research on pharmaceutical change of marketed Traditional Chinese Medicine (Trial)	0.67	1	0.25	1	0.5	0.67	0.67	0.5	0.6	5.86
Technical guidelines for pharmaceutical research of Traditional Chinese medicine compound Preparations managed according to the directory of ancient classic Prescriptions (Trial)	0.67	1	0.25	1	0.5	0.67	0.67	0.5	0.6	5.86
Guidelines for the writing of theoretical application materials for New compound preparations of Traditional Chinese Medicine (Trial)	0.67	1	0.25	1	0.5	0.67	0.67	0.5	0.6	5.86
Guidelines for writing instructions of traditional Chinese medicine compound preparations with ancient famous prescriptions (Trial)	0.67	1	0.25	1	0.5	0.67	0.67	0.5	0.6	5.86
Guiding principles for the comprehensive analysis of the effectiveness of drug clinical research (Trial)	0.67	1	0.25	1	0.5	0.67	0.67	0.5	0.6	5.86
Technical guidelines for the study of samples for toxicological research of New Chinese Medicine (Trial)	0.67	1	0.25	1	0.5	0.67	0.67	0.5	0.6	5.86
Technical guidelines for clinically independent drug studies	0.67	1	0.25	1	0.5	0.67	0.67	0.5	0.6	5.86
Guidelines for the randomization of drug clinical trials (Trial)	0.67	1	0.25	1	0.5	0.67	0.67	0.5	0.6	5.86
Communication guidelines based on the “three in One” evidence system for registration and review (for trial implementation	0.67	1	0.25	1	0.5	0.67	0.67	0.5	0.6	5.86
Guiding principles for clinical research and development of new drugs of traditional Chinese medicine compound preparations based on human experience (trial)	0.67	1	0.25	1	0.5	0.67	0.67	0.5	0.6	5.86
Technical guidelines for protocol change during drug clinical trials (Trial)	0.67	1	0.25	1	0.5	0.67	0.67	0.5	0.6	5.86
Technical guidelines for the Study of Clinical dependence of Drugs (trial)	0.67	1	0.25	1	0.5	0.67	0.67	0.5	0.6	5.86
Technical guidelines for research on drugs with the same name and the same prescription (Trial)	0.67	1	0.25	1	0.5	0.67	0.67	0.5	0.6	5.86
Guiding principles for the blinding of drug clinical trials (Trial)	0.67	1	0.25	1	0.5	0.67	0.67	0.5	0.6	5.86
Special Regulations on the Registration and Administration of Traditional Chinese Medicine in 2023	0.83	1	0.25	1	1	0.83	1	1	1	7.91
Measures on Further Strengthening the scientific supervision of Traditional Chinese Medicine to Promote the Inheritance and Innovation of Traditional Chinese Medicine in 2023	0.83	1	0.25	1	1	0.83	1	1	0.8	7.71
Measures for the Quality Supervision and Administration of Drug Business and Use 2023	0.83	1	0.25	1	1	0.67	1	1	0.6	7.35
Measures for the Administration of Drug Standards 2023	0.83	1	0.25	1	1	0.83	0.67	1	0.6	7.18
Measures for the Supervision and Inspection of Drug Clinical Trial Institutions (Trial Implementation) in 2023	0.83	1	0.25	1	1	0.83	0.67	0.75	0.6	6.93
Communication guidelines for drug registration applications supported by real-world evidence (trial implementation)	0.67	1	0.25	1	0.5	0.67	0.67	0.5	0.8	6.06
Guidelines for summary analysis and reporting of safety information during drug clinical trials (trial)	0.67	1	0.25	1	0.5	0.67	0.67	0.5	0.6	5.86
Technical guidelines for research on the preparation of drugs for clinical trials of new traditional Chinese Medicine (Trial)	0.67	1	0.25	1	0.5	0.67	0.67	0.5	0.6	5.86
Technical guidelines for pharmaceutical research of other Traditional Chinese medicine compound Preparations derived from ancient classical formulas (Trial implementation)	0.67	1	0.25	1	0.5	0.83	0.67	0.75	0.8	6.47
Technical guidelines for pharmaceutical research of new compound preparations of traditional Chinese Medicine based on human experience (trial)	0.67	1	0.25	1	0.5	0.67	0.67	0.5	0.6	5.86
Average value	0.661272727	0.942	0.25	0.963636364	0.557818182	0.643393939	0.677818182	0.547515152	0.614545455	5.858

With the advancement of China’s pharmaceutical industry, the focus of new Chinese medicine registration has shifted from the general provisions stated in P1, P23, P25, P113, and P114 of the Drug Administration Law of the People’s Republic of China, as well as the Law of the People’s Republic of China on TCM and Measures for the Administration of Drug Registration to specific implementation plans (P26, P37, P47, P83, P100, and P117). For instance, this includes guidelines such as those for Clinical Research on New Chinese Medicine, supplementary regulations concerning Registration Administration for Chinese Medicine, simplified registration and approval administration regulations for Ancient Classic Famous Chinese Medicine Compound Preparations and special regulations regarding Registration Administration for Chinese Medicine. The introduction provided by P26 focuses primarily on clinical trial research related to new Chinese medicine while clearly highlighting key aspects associated with such research. P37 further refines and clarifies the requirements for the registration management of Chinese medicine, thereby enhancing the emphasis on relevant provisions for registering and applying Chinese medicine compound preparations. This positively contributes to promoting research, development, and production of new Chinese medicine. It is proposed that the development of new TCM should adhere to TCM theory, accentuate TCM characteristics, prioritize clinical practice as a foundation, and ensure safety, effectiveness, and quality control of TCM. The supplementary provisions in question further elucidate the relevant requirements for registering and applying TCM compound preparations. The provisions on P47 clearly stipulate that the application for listing ancient classic famous prescription preparations meeting relevant requirements should only include pharmaceutical and non-clinical safety research data. This significantly shortens the R&D cycle of new drugs, reduces enterprise research and development risks and costs, and to some extent promotes the R&D of ancient classic famous prescription preparations. The P83 highlights the increasingly prominent status and distinctive characteristics of TCM, while China actively promotes the advancement of TCM.

**Figure 4:**
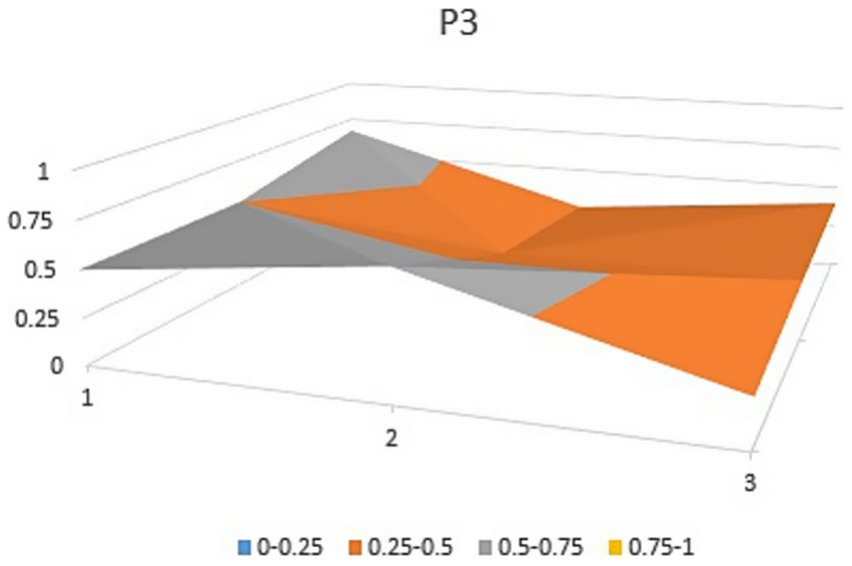
Bump plot of Policy P3.

**Figure 5:**
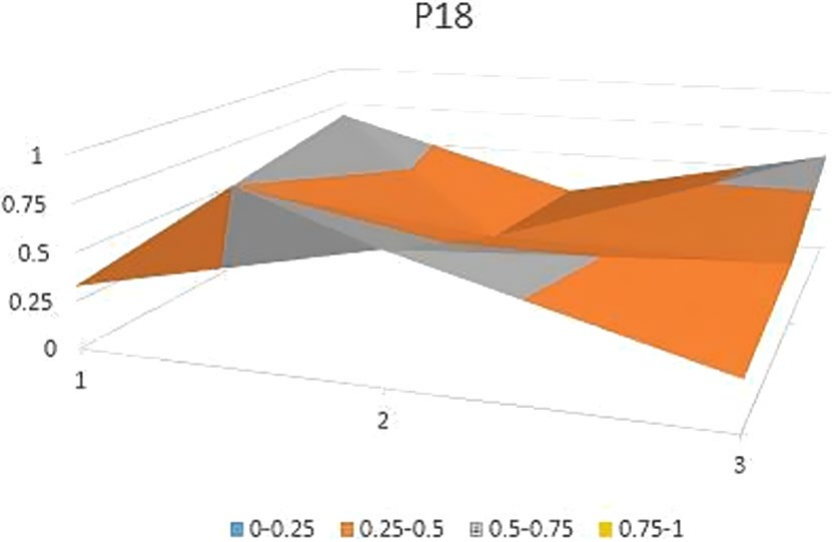
Bump plot of Policy P18.

**Figure 6:**
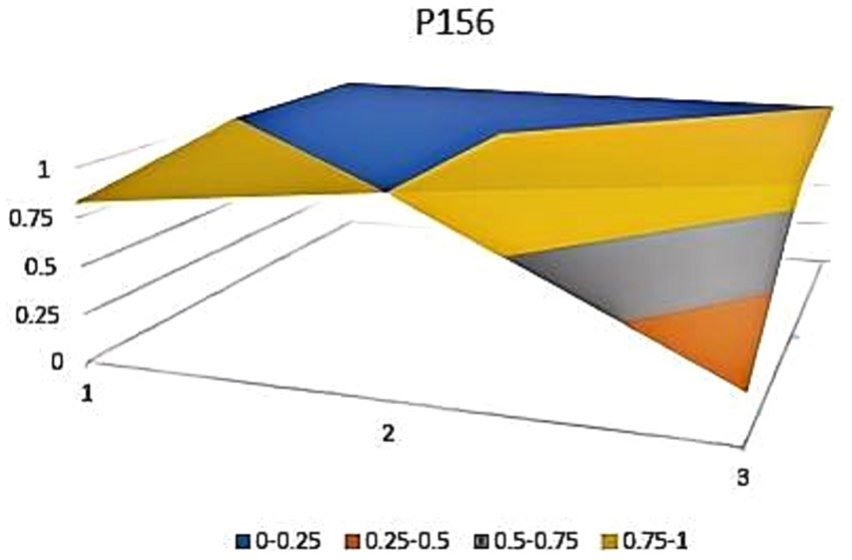
Bump plot of Policy P156.

**Figure 7:**
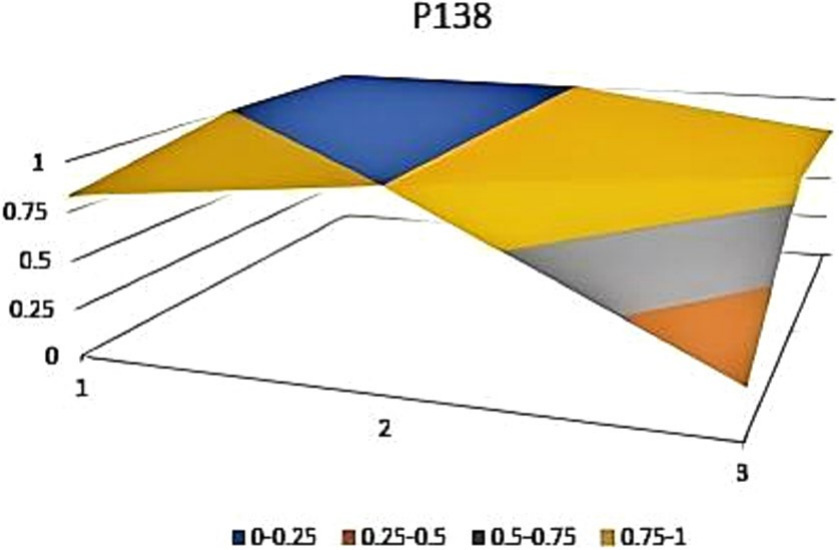
Bump plot of Policy P138.

The aforementioned observations collectively demonstrate the ongoing refinement of the TCM’s new drug registration policy formulation, as well as a shift toward an optimal policy that aligns with China’s national conditions and facilitates its implementation. Well-crafted policies and their effective execution will propel the advancement of new drug registration while elucidating strategic priorities for fostering innovation and development in TCM. Continual revisions and enhancements to regulations and standards are progressively closing the gap with other countries and regions. However, an analysis of recent years’ TCM registration and approval outcomes still highlights existing challenges in research, development, and registration management of TCM in China, warranting further attention and comprehensive discussion. In this study, P3, P18 with the lowest PMC index and P156 and P138 with the highest PMC index were selected to directly reflect the differences in China’s new drug registration policies. The PMC matrix is used to generate the PMC surface corresponding to the selected strategy. Convex surfaces indicate higher scores for each major variable, while concave surfaces indicate lower scores. The PMC index of P3 and P18 was 3.9 and 3.93, respectively, ranking the last and second among 165 policies for the registration of new Chinese medicines (Figures 4, 5). P3 was promulgated by the State Medical Products Administration when the concept of drug registration was first introduced in China. The idea of drug registration is still in the exploratory stage and can only be learned from countries that have already established a sound drug registration system. Therefore, there is a big gap in the construction of a new TCM registration system. P156 and P138 policies have the highest PMC index scores of 7.91 and 7.71 among the selected policies, and the two policies were promulgated in 2023 and 2021, respectively, which coincided with China’s formal entry into a new era of drug review and approval system (Figures 6, 7). During this period, the definition of a new drug was revised to include “drugs not sold within or outside China.” According to the originality and novelty of their material structure, new drugs can be divided into innovative drugs or improved drugs. This method takes into account the unique characteristics of the Chinese medicine system, and actively integrates foreign drug classification, so as to distinguish biological products from chemical drugs. It reflects the latest trend of new drug development at this stage. This is the ups and downs of China’s drug review and approval system for decades, has accumulated rich experience, efforts to reform, and innovation, and continues to move toward the comprehensive scientific and technological review process of declared drugs, with a completion rate of more than 95%. Review systems are constantly being developed and strengthened to align closely with international standards.

#### 4.2.2 Specific evaluation of TCMRPs

Revised sentence: By utilizing the evaluation index system and the PMC index model, we employ calculations to determine the multiple input and output matrix of 165 TCMRPs, along with their respective first-level index values and corresponding PMC indices for each policy. Furthermore, through the integration of the initial score table of secondary indices and PMC surface diagram in conjunction with specific TCMRPs, we focus on analyzing the consistency among TCMRPs indicators from various perspectives, thereby facilitating a comprehensive identification and analysis of their characteristics.

##### 4.2.2.1 Nature of policy (X1)

The mean values of the 165 secondary indexes X1-1, X1-2, X1-3, X1-4 and X1-5 for TCMRPs were recorded as 0.541, 0.935, 0.907, 0.943, 0.762, and 0.784, respectively. More than 70% of the registration policies for TCM new drugs encompass multiple policy orientations. During the process of designing policies for Chinese medicine’s new drug registration, the government places greater emphasis on supervising Chinese medicine’s new drug registration by providing guidance and describing registration conditions. Some policies include plans specifically tailored toward Chinese medicine registrations. However, state support in this regard is insufficient. There are limited predictive tendencies regarding future registrations of TCM new drugs. Nevertheless, the recently issued registration policy for TCM has significantly strengthened these predictive tendencies while also demonstrating support for new Chinese medicine registrations but with a weakened role in supervision.

##### 4.2.2.2 Prescription of policy (X2)

**Figure 8:**
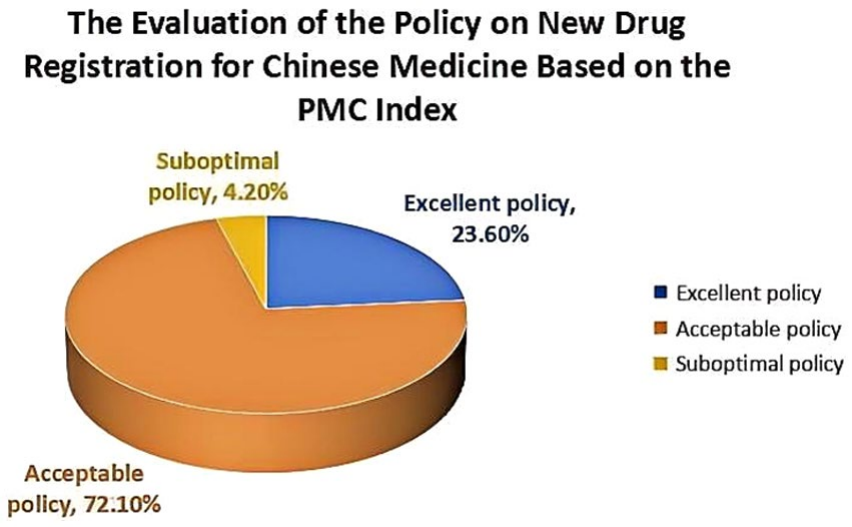
The Evaluation of the Policy on New Drug Registration for Chinese Medicine Based on the PMC Index.

Among the 165 policies examined in this study, 136 (82.4%) were identified as long-term policies, while 29 (17.6%) included medium-term policy indicators, none of them were classified as short-term policies within a one-year timeframe (Figure 8). This observation highlights the state’s commitment to long-term planning for TCM registration when formulating policies. The formulation of registration standards for new Chinese medicine began in 1985, reflecting efforts to establish and continuously enhance the registration system for new Chinese medicine in China. It is noteworthy that most of the non-long-term planning policies are supplementary regulations and management norms, which serve as regulatory documents supporting the registration standards for new TCM products. The average PMC index of X2 is calculated at 0.942, indicating a clear intention by the state to develop a long-term plan for new Chinese medicine registration with an expectation of ongoing optimization and improvement.

##### 4.2.2.3 Institution of publication (X3)

From the perspective of X3’s publishing agency, the average value of the first-level indicator X3 is 0.25. All policies are formulated by national authorities. Due to China’s unique national conditions, state organs coordinate with provincial new drug registration agencies to ensure compliance with national laws and regulations, as provincial regulatory authorities lack the ability to formulate their own legislation. Therefore, out of the 165 collected policies, all were issued by the state, highlighting the country’s emphasis on registering new drugs in TCM and promoting standardized policy patterns for such registrations nationwide. This unification establishes a consistent national standard for registering new Chinese medicine and facilitates their production and use throughout the country, thereby fostering circulation and development within China’s comprehensive plan for its TCM industry.

##### 4.2.2.4 Target of policy (X4)

The policy object encompasses all policies, including X4-1, X4-2, and X4-3. Among these, the administration of X4-1 is bestowed by the national administration upon provincial administrations as a supervisory authority. X4-2 represents enterprises that initiate the new Chinese medicine registration policy, indicating its primary objective of establishing standards for enterprises. Both X4-3 (hospitals) and X4-4 (testing institutes) provide services related to Chinese medicine’s new drug registration. Hospitals primarily serve for clinical testing in this process and offer facilities and personnel for conducting trials. Testing institutes mainly determine the material components of Chinese medicine new drugs and can proceed with clinical testing at hospitals once their materials are qualified. In summary, each institution assumes specific responsibilities and functions within the comprehensive system of new Chinese medicine registration, completing registrations through mutual coordination among various functional departments.

##### 4.2.2.5 The purpose of policy (X5)

The average PMC index of each policy in the X5 (policy function) is 0.558, indicating a favorable performance. Among the 165 selected policies, there is a slight inclination toward optimization for X5-1, and only one policy, “Opinions on Reforming the Review and Approval System of Drugs and Medical Devices” (2015), demonstrates a tendency to optimize the product structure of X5-5. Most of these policies fall under X5-2 (which establishes standards), X5-3 (which optimizes procedures), X5-4 (which encourages innovation) and X5-6 (which improves the system). This data reveals that China’s current approach to new drug registration for TCM involves formulating standards, optimizing procedures, and improving the system. Encouraging innovation is proposed in “Opinions on Promoting the Inheritance, Innovation and Development of TCM.” To promote innovation and development within TCM culture, China has begun emphasizing encouragement and innovation within new Chinese medicine.

##### 4.2.2.6 Content of the policy (X6)

The secondary indicators of the X6 (policy content) primarily encompass X6-1 (technical guidance), X6-2 (prioritized resource allocation), X6-3 (shortened evaluation time limits), X6-4 (application conditions), X6-5 (application pathways), and X6-6 (registration verification and inspection). Among them, laws and regulations such as the Drug Administration Law of the People’s Republic of China, the TCM Law of the People’s Republic of China, and the Opinions on Promoting the Inheritance, Innovation and Development of TCM provide policy guidance opinions without explicitly elaborating on new Chinese medicine registration standards. Instead, they offer detailed information regarding application conditions, application pathways, registration verification procedures and inspections. The technical guidance documents include “New Drug Approval Measures,” “Chinese Medicine New Drug Pharmaceutical Research Guide,” “Chinese Medicine New Drug Clinical Research Guiding Principles,” “Chinese Medicine Injection Research Guiding Principles” among others. The average PMC index for the policy content of X6 is 0.643 which indicates a satisfactory level within an acceptable range, however there is still room for improvement. Therefore it is necessary for the government to continuously optimize and enhance these policies with a primary focus on prioritizing resource allocation (X6-2).

##### 4.2.2.7 Policy approach (X7)

The incentive methods of the policy are categorized into three types: X7-1 (compulsory type), X7-2 (service type), and X7-3 (incentive type). These three measures consist of the Notice on Several Issues concerning Drug Approval and Administration (1992), the Measures for Drug Registration Administration (2020), and the Special Provisions on the Registration and Administration of TCM (2023). Most policy documents only include two incentive measures, primarily focusing on compulsory and service aspects, with no specific incentives outlined. Apart from the aforementioned documents, texts incorporating incentive policies encompass the Regulations on Simplified Registration and Approval Management of Compound Preparations of Ancient Classical Chinese Medicine (2018) as well as the Opinions on Promoting the Inheritance and Innovation of TCM. According to the Regulations on Simplified Registration and Approval Administration of Compound Preparations of Ancient Famous Prescriptions in TCM (2018), clinical trials can be exempted during TCM registration. Considering that new drug research, development, and clinical trials entail substantial time and capital investment for pharmaceutical enterprises in registering new Chinese medicine products, utilizing ancient classic prescriptions significantly reduces investment costs for these enterprises. Encouraging pharmaceutical companies to develop ancient classic prescriptions not only diminishes research expenses but also effectively explores TCM resources. This approach contributes to preserving TCM culture through inheritance and innovation while safeguarding its overall development.

##### 4.2.2.8 The approach to motivation (X8)

The incentive methods encompass X8-1 (procedure simplification), X8-2 (registration subsidy), X8-3 (intellectual property protection), and X8-4 (supervision and evaluation). Based on the collected policies prior to 2008, China’s emphasis on intellectual property rights protection was lacking, with no specific policy addressing registration subsidies. This suggests that in 2008, China did not perceive incentives such as registration subsidies and intellectual property protection as effective means of stimulating innovative research and development of new TCM. However, from 1985 to 2023, all policies have consistently streamlined procedures for the registration of new Chinese medicine products, facilitating rapid availability of qualified new drugs while expediting their market value realization. Furthermore, comprehensive supervision and evaluation measures were implemented to encourage pharmaceutical enterprises’ research and development efforts through reasonable oversight. Post-2008 policies gradually shifted focus from procedure simplification toward prioritizing intellectual property protection by employing diverse approaches to incentivize the registration of new TCM.

##### 4.2.2.9 Scientificity of policy (X9)

The average PMC index of the policy’s scientific nature was 0.615, which falling within an acceptable range. The evaluation of the policy’s scientific nature was conducted based on X9-1 (with sufficient foundation), X9-2 (with comprehensive content), X9-3 (with detailed measures), X9-4 (with clear division of labor), and X9-5 (with explicit responsibilities and rights). Approximately 85.7% of the policies had comprehensive content, clear division of labor, and detailed measures. 71.4% of the policies met all four secondary indicators except for explicit responsibilities and rights, while only 32.1% of the policies fulfilled all aforementioned indicators simultaneously. This indicates that China’s policy scientificity needs optimization in terms of clarifying responsibilities and rights as well as confirming their identification further. Notably, the Measures for Drug Registration Administration (2020) clearly stipulates the responsibilities and rights of drug registration supervisors and persons in charge. The differentiation in responsibilities and rights is beneficial for the subsequent implementation of new Chinese medicine registration system by defining departmental roles effectively, ensuring the rational exercise of power, promoting a high-quality and safe registration process for the new Chinese medicine products while fostering innovation and development within TCM.

## 5 Conclusion and implications

### 5.1 Conclusion

This study assessed and analyzed the consistency of the PMC index in Chinese medicine new drug registration policies from 1985 to 2023 through text mining. Building on previous research, we identified key characteristics of TCMRPs and found that, while these policies have progressively improved, certain limitations persist. We examined each secondary index’s PMC values separately and used these values to propose recommendations for enhancing TCM new drug registration policies. Our study represented the first quantitative exploration of Chinese medicine new drug registration policy consistency, filling a gap in existing literature.

Through the analysis of the high-frequency terms in the new drug registration policy for traditional Chinese medicine, it is evident that an effective registration system for new Chinese medicines should be established based on national policies and in collaboration with various national authorities such as The National Medical Products Administration (NMPA), the State Administration of TCM, and other relevant state agencies. The provincial drug control institute should be served as the testing institution while hospitals can function as clinical trial institutions, forming an integrated framework. In terms of innovation, a thorough exploration of ancient Chinese traditional medicine culture rooted in classical prescriptions is essential. Enhancing technical requirements and refining the registration system for new drugs derived from TCM are shared priorities within China’s new drug registration policy.

In this study, the PMC index model was utilized to systematically assess the TMCRPs. The findings revealed that the average PMC index of 165 TCM new drug registration policies was 5.858. The total number of policies is divided into three categories: 39 policies (23.6% of all policies) are classified as excellent, 119 policies (72.1% of all policies) are considered acceptable, and there are 7 poor policies (4.2% of all policies). The average primary variables for policy issuing institution X3 and incentive method X8 in TCMRPs are relatively low. Based on China’s national conditions, it remains imperative to uphold the state as the primary authority for policy formulation, enhance communication between the central and provincial departments, and bolster the efficacy of policy implementation by relevant provincial entities in order to optimize implementation efficiency. Furthermore, the State should devise tailored incentives that align with specific national requirements while retaining flexibility to adapt these measures accordingly, a marginal escalation in incentive intensity could also be contemplated.

Among the 165 selected policies, the first-level indicators exhibiting relatively favorable performance include policy nature (X1), policy object (X4), policy function (X5), policy content (X6), policy mode (X7), and policy scientificity (X9). Although the average value stands at 0.693 and is considered satisfactory, it should be noted that these policies may overlook certain second-level indicators as they often only address a subset of them. For instance, in relation to X1, the aspects such as prediction, guidance, description, supervision planning and support are not adequately addressed within the policies, with prediction and support being mentioned only in a limited number of documents. Therefore, there is a need to strengthen predictive capabilities within policymaking while also providing support for the registration of new TCM. Additionally, when considering the functions outlined in X5 policies should prioritize guiding development efforts and strive toward fostering TCM culture. In terms of product structure optimization should be emphasized along with clarifying product independence and innovation while recognizing their inherent value so as to generate greater economic and medical benefits. Lastly but importantly, the commendable progress made in enhancing the construction of new TCM registration systems necessitates continued commitment from authorities to establish an impeccable system that evolves alongside societal advancements. In terms of policy content X6, there is also a need for a reallocation of resources to prioritize and unite multiple government departments for coordinated development, rationalization, and efficient utilization of limited resources such as hospitals and pharmaceutical laboratories. Reasonable allocation of these resources can effectively reduce review time limits, minimize resource waste and vacancies. Additionally, we should attach great importance to the protection of intellectual property rights (X8-3) as intangible capital that brings substantial profits to enterprises and contributes to national wealth. Neglecting the safeguarding of intellectual property rights may result in other countries preemptively acquiring development rights over ancient Chinese classic famous parties, which hinders the preservation of TCM. Therefore, it is crucial to strengthen the inheritance and innovation of TCM culture. In terms of policy scientificity, it is necessary to enhance measures refinement by establishing detailed regulations and standards at every step of new Chinese medicine registration process ensuring its safety and effectiveness. Clear allocation of responsibilities and rights guarantees that all parties have their due entitlements while distinguishing their respective obligations so that when issues arise, there are legal grounds supported by evidence with assigned accountability.

In summary, while the majority of policies governing the registration of TCM new drugs exhibit sound policy design and consistency, a meticulous examination of the sample of outlier policies reveals potential weaknesses and deficiencies in the policy framework for registering TCM that warrant our attention and analysis. Taking into account the findings from this study’s PMC index calculations and specific policy content, we can broadly identify shortcomings in the following areas: (1) Currently, China lacks a comprehensive evaluation system for innovative TCM, which poses numerous challenges during their application process. (2) The research and development of innovative TCM faces various technical bottlenecks such as extraction, purification, and quality control of active ingredients. These technical challenges constitute one of the primary factors that have impeded the development of novel TCM, thereby influencing both the number of applications submitted and the approval rates for new drug candidates. (3) The innovation, research, and development of new Chinese medicines require a substantial number of skilled professionals. However, talent protection is not addressed adequately in the analyzed policies. Presently, there is a shortage of talent within China’s TCM field hindering innovation efforts as well as research & development activities related to new Chinese medicines. (4) The TCM new drug policy in China gives limited attention to specific policy areas and often relies on coercive measures such as the establishment of laws, regulations, and standards. However, it overlooks financial support and other economic incentives. (5) Only in the past 5 years has China started paying attention to exploring ancient classical prescriptions. However, there has been limited exploration so far. Insufficient emphasis has been placed on preserving China’s 5,000-year-old traditional medicine culture.

### 5.2 Implications

Based on the aforementioned analysis, we have identified that this study holds significant implications for optimizing the structural design in TCMRPs.

It is imperative to thoroughly examine and optimize the combination and relationship of various elements within the registration policy of TCM. The registration process for new Chinese medicine serves as a cornerstone of the Chinese government’s commitment to supporting and promoting sustainable and healthy development within TCM. The policy content outlines key aspects of registering new Chinese medicine, encompassing registration standards, procedures, validity, scope, ownership of responsibilities and rights, all playing a pivotal role in effective policy implementation. Therefore, in order to achieve our objective of ensuring safe, effective, and expeditious registration of new TCM while fostering innovation and development within TCM field it is essential to re-evaluate policy elements along with their respective roles. Scientifically sound optimization should be conducted by addressing any deficiencies present within the current policies. (1) It is crucial to establish a comprehensive policy framework consisting of a solid foundation-target-path approach followed by normative strategies that clearly define and rationalize interrelationships between different policy elements. (2) The policy-making process should follow these key steps: First, establish clear policy objectives. Second, develop evidence-based policies through rigorous scientific methods. Third, identify policy priorities through systematic analysis of implementation trends. Fourth, strengthen regulatory oversight of these priority areas. Finally, incorporate predictive modeling to enhance policy foresight. (3) The Policy formulation should adhere to the principle of coherence to prevent redundancy and conflicts in content and nature among different policies. Taking the registration process for new Chinese medicine as an example, various policies establish specific procedures for such registration. When all procedures strictly adhere to these well-defined policies, a comprehensive and scientific system for registering new Chinese medicine can be established. (4) The registration policy for TCM needs to be approached from multiple perspectives that represent different interests. From an enterprise standpoint, it is desirable that the policy supports research and development efforts. From a government perspective, it is important that the policy facilitates regulation. Therefore, we need to formulate policies that incentivize enterprises to engage in research and production while fostering innovation enthusiasm, simultaneously strengthening supervision measures are necessary to ensure safety and efficacy standards are met.

The improvement of safeguards and incentives is also necessary. According to this study, the low PMC index of certain TCM new drug registration policies can be attributed to the inadequate consideration of insurance incentives in policy design. However, the registration process for new TCM is a comprehensive and intricate system that involves multiple parties. Different departments should fulfill their respective roles and establish a well-functioning registration system. Therefore, the following changes need to be made: firstly, strengthen the responsibilities and potential involvement of various ministries in the registration system for TCM, enhance coordination among NMPA, provincial food and drug administrations, testing institutions, hospitals, and other organizations, form functional integration among different management systems based on national development plans for new Chinese medicine registrations, provide scientific and effective supervision as well as support for registering new TCM. Secondly, it is essential to reinforce guarantee incentive measures such as institutional guarantees, economic guarantees, organizational guarantees, and technical guarantees, boost departmental enthusiasm toward registering new Chinese medicines, lay a foundation for innovation and development within TCM, increase willingness to take action in order to achieve policy objectives.

### 5.3 Limitations and further works

Based on the PMC index model, this study analyzed the advantages and disadvantages of the registration policy of TCM from a policy-making perspective, providing novel ideas and theoretical foundations for future evaluation of such policies. However, there are still some limitations in this study. Firstly, the determination of primary and secondary variables involves certain subjectivity. To obtain more objective and scientific data for policy evaluation, additional theoretical support and technical assistance such as big data mining and grounded theory should be considered for optimization purposes. Secondly, this article does not examine the changes in the number of new drug approvals therefore cannot determine whether policy enhancements have positively influenced these changes. Lastly, the present article lacks comprehensive industrial data, and it remains unclear whether the revised registration policies for new TCM have effectively facilitated the development of the TCM industry. In future research, we propose incorporating both drug approval statistics and TCM innovation industry metrics into the analytical framework to systematically evaluate potential policy impact on regulatory outcomes and sectoral development.

## Data Availability

The original contributions presented in the study are included in the article/Supplementary material, further inquiries can be directed to the corresponding author/s.
